# Development of Volatile Compounds during Hydrolysis of Porcine Hemoglobin with Papain

**DOI:** 10.3390/molecules23020357

**Published:** 2018-02-08

**Authors:** Kathrine Holmgaard Bak, Mikael Agerlin Petersen, René Lametsch, Erik T. Hansen, Jorge Ruiz-Carrascal

**Affiliations:** 1 Department of Food Science, University of Copenhagen, Rolighedsvej 26, 1958 Frederiksberg C, Denmark; map@food.ku.dk (M.A.P.); rla@food.ku.dk (R.L.); jorgeruiz@food.ku.dk (J.R.-C.); 2 Danish Crown Ingredients, Flæsketorvet 41, 1711 Copenhagen V, Denmark; eth@dcingredients.dk

**Keywords:** hydrolysis, enzyme, enzymatic hydrolysis, hemoglobin, papain, aroma, volatile compounds

## Abstract

There is a growing market for the use of hydrolysates from animal side-streams for production of high-protein supplements. However, there can be issues with development of off-flavors, either due to the raw material in question or due to the hydrolysis process itself. This study examined the development of volatile compounds during hydrolysis of hemoglobin. Briefly, porcine hemoglobin was hydrolyzed by 0.5% papain for up to 5 h, and the development of volatile compounds was analyzed via gas chromatography-mass spectrometry. The results showed that there was significant development of a number of volatile compounds with time, e.g., certain Maillard reaction and lipid oxidation products, which are likely candidates for the aroma development during hydrolysis. Furthermore, it was shown that development of a number of the volatiles was due to the hydrolysis process, as these compounds were not found in a control without enzyme.

## 1. Introduction

Porcine blood is a significant by-product from the slaughter industry, reported to make up 6–7% of the lean meat content of the carcass [1]. Whereas blood was previously often used in dishes such as blood sausage, it is now rarely used in household cooking and, instead, used for other purposes, mainly animal feed [1,2] and fertilizer [2,3,4]. Increasing the use of by-products from animal slaughter can significantly improve both profit for the meat industry and the sustainability of animal production [2].

At the slaughterhouse, blood is usually divided into its two fractions, plasma and the cellular fraction, the latter mainly consisting of hemoglobin [5]. The plasma fraction is currently being used in food products for human consumption, e.g., as an emulsifier [5,6]. This is rarely the case for hemoglobin, partly due to the color, but also due to the strong flavor, which in many products may be considered an off-flavor [7,8], and due to the prooxidant effect of heme [9]. However, due to the high protein content [7] and high lysine content in particular [1,10], there is a large potential for the use of hemoglobin in different types of food products, which are low in lysine.

One way of adding the hemoglobin-derived protein to food products is by adding them in the form of hydrolysates, which may provide additional health benefits [11,12,13,14,15]. Additionally, by hydrolyzing hemoglobin, the heme group can easily be removed, reducing the intense, metallic taste as well as the prooxidant activity [16]. However, hemoglobin hydrolysates, on top of showing bitter taste, which is characteristic for protein hydrolysates in general, show off-flavors that make the product unacceptable for many food applications.

In order to optimize the flavor of hemoglobin hydrolysates, it is crucial to establish whether such off-flavors are already formed during the acquisition and storage of the cellular fraction, or if they are formed during the hydrolysis process. A first approach to tentatively address the origin of this off-flavor is to identify the volatile compounds present in the raw material as well as those formed during the hydrolysis process. Previous studies on different types of hydrolysate have shown that both the raw material and the conditions during the hydrolysis may influence the profile of volatile compounds formed [17,18,19]. In these studies, compounds from lipid oxidation and from Maillard reactions have been found. While some of those compounds may actually contribute positively to the final aroma profile of the hydrolysates, others might constitute off-flavors, inhibiting the use of the hydrolysate in food applications.

Hence, the aim of this work was to determine the development of volatile compounds during hydrolysis of hemoglobin in order to address to what extent these compounds are a result of the hydrolysis itself or already present in the raw material. If possible, candidates for off-flavor-development during hydrolysis of hemoglobin will be identified.

The results showed that during hydrolysis of hemoglobin, there was a significant development of a number of volatile compounds over time, e.g., certain Maillard reaction and lipid oxidation products, which are likely candidates contributing to the aroma of the final product. Furthermore, it was shown that the development of a number of the volatiles was due to the hydrolysis process itself, as these compounds were not found in a control undergoing the same processing conditions but without enzyme.

## 2. Results and Discussion

Table 1 shows the yield, defined as percentage of the total dry matter content of the hydrolysate recovered in the protein fraction, at each time point during hydrolysis. Not surprisingly, yield appeared to increase with time of hydrolysis, but particularly in the last two hours of hydrolysis, whereas no change in yield was apparent between 1 h and 3 h.

When producing a protein hydrolysate, as high a yield as possible is generally desirable. But of course, the development and disappearance of both desirable and undesirable volatile compounds during the course of hydrolysis should be considered.

Table 2 shows the names and relative concentrations of the volatile compounds detected via gas chromatography-mass spectrometry (GC-MS). Among the 67 volatile compounds that were detected, 23 were found to change significantly in concentration during hydrolysis, and four exhibited an increase by a factor 4 or more. It is likely that some of these compounds could change the flavor of the hydrolysate, though it should be kept in mind that perceived aroma depends on factors such as concentration, threshold, and matrix [20].

The Strecker aldehydes 3-methylbutanal and benzeneacetaldehyde increased by factors 4.5 and 8 and have dry/green and hyacinth odors. 3-Methylbutanoic acid and hexanoic acid (cheesy and rancid odor descriptors) increased 15 and 42 times, respectively (all odor descriptors from [21]). Some of the mentioned compounds contribute with unpleasant/unwanted odors, but even more pleasant odors may be perceived as off-flavors, depending on the context in which the hydrolysate is used.

**Figure 1:**
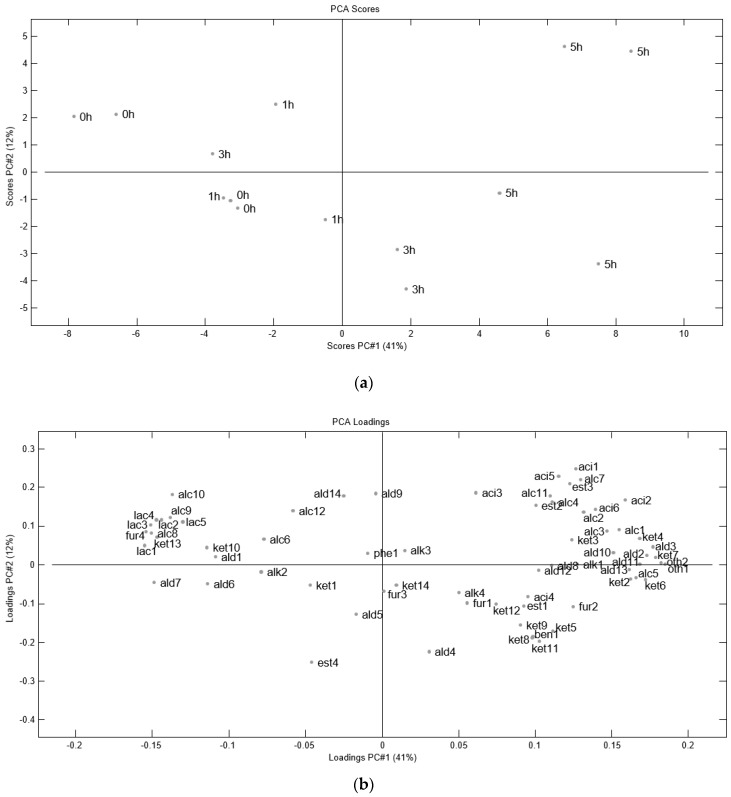
(**a**) Principal Component Analysis (PCA) score plot (minus outliers) for the effect of time on enzymatic hydrolysis of porcine hemoglobin with papain; (**b**) PCA loading plot showing the occurrence of volatile compounds during hydrolysis. Codes are equivalent to those found in Table 2.

Figure 1a shows the score plot from the Principal Component Analysis (PCA) on volatile compounds during hydrolysis of porcine hemoglobin. As expected, there is a clear effect of time, especially a clear separation between 0 h and 5 h on PC1, which explains 41% of the variance. The loading plot in Figure 1b shows how the occurrence of different volatile compounds changes during hydrolysis, since compounds to the left of the loading plot are present mainly at 0 h, whereas compounds to the right of the loading plot are present mainly after 5 h of hydrolysis.

Compounds that were found to increase with time of hydrolysis but were not found in the controls without enzyme (results not shown) include: 3-methyl-2-pentanone, benzoic acid, tetradecane, and 3-methylbutanenitrile. These compounds must therefore be a result of the enzymatic hydrolysis.

The most abundant compound was by far benzaldehyde, which also increased in concentration with time (Figure 1b, Table 2). Benzaldehyde and its derivatives have been described as compounds arising from Maillard reactions [22], although other routes of formation have been suggested, such as the oxidation of different toluenes and other hydrocarbons [23].

Compounds derived from the Strecker degradation of amino acids, such as 2- and 3-methylbutanal showed a clearly increasing trend throughout hydrolysis. This makes sense, since the substrates for the formation of such aldehydes, leucine and isoleucine respectively, together with different reducing sugars, are present in blood [8]. Moreover, carbonyl compounds from the oxidation of lipids [24] or proteins [25] have been demonstrated to promote the oxidative degradation of amino acids and yield the corresponding Strecker aldehydes.

Some of the compounds with a higher load on PC1 were methyl ketones (3-methyl-2-pentanone and 3-methyl-2-butanone) and 𝛼-diketones (e.g., 2,3-pentanedione and 2,3-butanedione), meaning that these compounds showed a trend towards increasing their levels throughout hydrolysis time. Both methyl-ketones and 𝛼-diketones have been described as arising as a consequence of microbial metabolism in different food products [26]. This could actually have been possible during hemoglobin hydrolysis, since there is no previous sterilization, and the incubation temperature (55 °C) would allow for the growing of some microorganisms.

Typical compounds from fatty acid autoxidation, such as straight chain aldehydes and alk-1-ols were found. Not all of these compounds showed the same trend. Hexanal, the most extensively used marker for lipid oxidation, showed a clear growing trend during the initial hours of hydrolysis, followed by a decrease to the original level. Other compounds showed only minor, statistically insignificant variation or even a decreasing trend in the case of octanal. The presence of these compounds is most likely a consequence of the oxidation of the phospholipids remaining in the red cell fraction given the conditions during hydrolysis, at 55 °C and with a massive amount of prooxidant heme.

In this study, the effect of hydrolysis time on the development of volatile compounds was analyzed. There are other factors, which could commonly change in an industrial setup, including the raw material and choice of enzyme(s) and its concentration, to name a few. The final produced hydrolysate should be tested in a real product for a realistic evaluation of the effect of hydrolysates on off-flavor and -odor. For instance, Meinert et al. [27] found that addition of hydrolysates of porcine and bovine origin increased the occurrence of off-flavors and -odors in saveloys compared to the control without hydrolysate.

## 3. Materials and Methods

### 3.1. Enzymatic Hydrolysis

Hemoglobin was provided by a commercial slaughterhouse and kept frozen in 50 mL tubes at −80 °C prior to use. The appropriate volume of hemoglobin was thawed overnight in the fridge. Four replicas of the hemoglobin were hydrolyzed as follows: Eighty mL of thawed hemoglobin was poured into a blue cap bottle (500 mL) and 160 mL of cold tap water was added. A magnet was added to each bottle for extra friction at the bottom of the bottles. The bottles were placed in a shaking water bath at 55 °C for temperature equilibration. Enzyme stock solution was made (four replica) by weighing out 1.4057 g of the endopeptidase papain (BSC Biochemicals, Hamme, Belgium) and adding 9.378 mL of cold tap water. When the temperature of the hemoglobin had reached 55 °C, 8 mL enzyme stock solution was added to each bottle for a final enzyme concentration of 0.5%.

In a similar way, four control samples without enzyme were produced by simply adding 8 mL of cold tap water instead of the enzyme stock solution.

At time points 0 h, 1 h, 3 h, and 5 h, a 40 mL sample was taken from each blue cap bottle and transferred to a 50 mL tube (for controls, only time points 0 h and 5 h) and immediately placed in a water bath set at 95 °C for 20 min of heat treatment to inactivate the enzyme. After cooling in an ice bath for 5 min, pH was lowered by adding 1400 µL 2 M sulfuric acid in order to precipitate the heme group. The tubes were then centrifuged at 3000 *g* for 15 min, and the supernatants (protein fraction) were poured into clean 15 mL tubes.

Dry matter content was determined for both the complete sample (taken prior to centrifugation) and the supernatant after overnight drying at 105 °C of a small sub-sample of each sample. The remaining sample was frozen (−20 °C) until further analysis.

### 3.2. Volatile Compound Analysis

#### 3.2.1. Dynamic Headspace Sampling

Samples were thawed overnight in the fridge (−4 °C). For each time point, 20 mL of each replica was transferred to a 100 mL gas washing flask and closed with a purge head. The samples were equilibrated to 37 °C in a circulating water bath and then purged with nitrogen (150 mL/min) for 60 min under magnetic stirring (200 rpm). The volatile compounds were collected on traps containing 200 mg of Tenax-TA with mesh size 60/80 (Markes International, Llantrisant, UK). Water was removed from the traps by dry-purging with nitrogen (100 mL/min) for 20 min.

#### 3.2.2. Gas Chromatography-Mass Spectrometry

The trapped volatiles were desorbed using an automatic thermal desorption unit (TurboMatrix 350, Perkin Elmer, Shelton, CT, USA). Primary desorption was carried out by heating the trap to 250 °C with a flow (50 mL/min) of carrier gas (H_2_) for 15.0 min. The stripped volatiles were trapped in a Tenax TA cold trap (30 mg held at 5 °C), which was subsequently heated at 300 °C for 4 min (secondary desorption, outlet split 1:10). This allowed for rapid transfer of volatiles to a gas chromatograph-mass spectrometer (GC-MS, 7890A GC-system interfaced with a 5975C VL MSD with Triple-Axis detector from Agilent Technologies, Palo Alto, CA, USA) through a heated (225 °C) transfer line. Separation of volatiles was carried out on a ZB-Wax capillary column 30 m long × 0.25 mm internal diameter, 0.50 µm film thickness. The column pressure was held constant at 2.3 psi, resulting in an initial flow rate of 1.4 mL/min using hydrogen as the carrier gas. The column temperature program was: 10 min at 30 °C, from 30 °C to 240 °C at 8 °C/min, and finally, 5 min at 240 °C. The mass spectrometer was operating in the electron ionization mode at 70 eV. Mass-to-charge ratios between 15 and 300 were scanned. Peak areas and mass spectra were extracted from the chromatograms using the PARAFAC2 based software PARADISe [28] and mass spectra were identified using the NIST05 database. Volatile compound identification was confirmed by comparison with retention indices (RI) of authentic reference compounds or retention indices reported in literature.

### 3.3. Data Analysis

#### 3.3.1. GC-MS

Principal component analysis was performed on autoscaled peak areas using the software Latentix 2.12 (Latentix Aps, Frederiksberg, Denmark). Analysis of variance was performed using JMP 13.0.0 (SAS Institute Inc., Cary, NC, USA) and least square means were compared by Student’s *t*-test (*p* ≤ 0.05) (JMP 13.0.0, SAS Institute Inc.).

#### 3.3.2. Yield

Yield was calculated for each time point as the percentage of total dry matter content of the hydrolysate recovered in the supernatant (protein fraction) after centrifugation.

## 4. Conclusions

The obtained results showed that during hydrolysis of hemoglobin, there was a significant development of 23 volatile compounds over time, e.g., certain Maillard reaction and lipid oxidation products, which are likely candidates contributing to the aroma of the final product. Furthermore, it was shown that the development of a number of the volatiles was due to the hydrolysis process itself, as these compounds were not found in a control having undergone the same processing conditions, only without enzyme. The final produced hydrolysate should be tested in a real product for a realistic evaluation of the effect of the hydrolysate on off-flavor and -odor.

## Figures and Tables

**Table 1:** Yield for each time point of the hydrolysis.

Time (h)	Yield (%)	SD (%)
0	59.8	±0.2
1	65.1	±0.2
3	64.9	±6.1
5	73.7	±1.2

**Table 2:** Relative concentrations as peak area/1000 of volatile compounds detected during hydrolysis.

Code ^1^	Compound	0 h ^2^	1 h	3 h	5 h	Retention Index		
						Exp.	Auth. Std.	Literature
alc1	2-Methyl-1-propanol	33	34	41	53	1100	1100	
alc2	Butanol	12	13	15	28	1165	1165	
alc3	2-Methylbutanol	34	53	68	147	1231		1158–1244
alc4	3-Methylbutanol	138	128	116	463	1232	1230	
alc5	3-Methyl-3-buten-1-ol	39 ^c^	47 ^b^	44 ^b, c^	52 ^a^	1268		1221–1277
alc6	1-Pentanol	37	40	40	33	1274	1274	
alc7	1-Hexanol	28 ^b^	9 ^b^	30 ^b^	295 ^a^	1372	1372	
alc8	1-Heptanol	55	43	39	17	1471	1471	
alc9	1-Octanol	63	54	46	32	1573	1573	
alc10	*Z*-10-Pentadecen-1-ol	33	31	11	15	1676		
alc11	Phenylethyl alcohol	1	1	6	190	1936	1936	
alc12	Dodecanol	25	36	1	15	1992		1919–1984
ald1	2-Methylpropanal	296 ^a^	226 ^b^	324 ^a^	206 ^b^	749		770–834
ald2	2-Methylbutanal	192 ^c^	242 ^b^	260 ^b^	353 ^a^	907	913	
ald3	3-Methylbutanal	914 ^d^	1406 ^c^	2029 ^b^	4109 ^a^	917	917	
ald4	Pentanal	133 ^b^	197 ^a^	184 ^a^	153 ^ab^	983	983	
ald5	Hexanal	791 ^b^	1427 ^a^	1508 ^a^	844 ^b^	1083	1088	
ald6	Heptanal	125	139	138	87	1192	1192	
ald7	Octanal	209 ^a^	234^a^	191 ^a^	107 ^b^	1303	1311	
ald8	Nonanal	265 ^b^	337^ab^	453 ^a^	440 ^a^	1406	1403	
ald9	(*E*)-2-Octenal	11	15	18	14	1441		1393–1467
ald10	Decanal	59 ^b^	55 ^b^	83 ^b^	160 ^a^	1510	1511	
ald11	Benzaldehyde	21,821 ^d^	38,502 ^c^	46,420 ^b^	53,809 ^a^	1530	1539	
ald12	(*E*)-2-Nonenal	15^c^	21 ^b, c^	32 ^a^	27 ^ab^	1549	1551	
ald13	Benzeneacetaldehyde	27^c^	51 ^c^	112 ^b^	222 ^a^	1650		1592–1684
ald14	(*E*)-2-Decenal	22	26	22	22	1660		1592–1682
est1	Ethyl acetate	74	49	86	102	864		850–914
est2	Hexyl acetate	0	1	2	223	1288	1293	
est3	Ethyl octanoate	1	1	1	52	1446	1445	
est4	Ethyl decanoate	3	3	3	2	1651	1651	
fur1	2-Pentylfuran	51 ^d^	111 ^b^	137 ^a^	89^c^	1243		1193–1258
fur2	1-(2,4-Dimethyl-furan-3-yl)-ethanone	17	25	109	137	1586		
fur3	2-Furanmethanol	2	17	2	3	1671		1613–1698
fur4	5-Ethyldihydro-2(3*H*)-furanone	83	65	59	2	1719		
ket1	2-Butanone	979	981	1051	923	887	906	
ket2	3-Methyl-2-butanone	985^c^	1746 ^b^	1803 ^b^	2122 ^a^	929		918–989
ket3	3,3-dimethyl-2-butanone	88	95	94	105	948		969–986
ket4	2,3-Butanedione	1665 ^c^	2079 ^b, c^	2404 ^b^	3166 ^a^	985	985	
ket5	4-Methyl-2-pentanone	25	37	60	73	1006		993–1040
ket6	3-Methyl-2-pentanone	512 ^c^	731 ^b^	751 ^b^	889 ^a^	1014	1001	
ket7	2,3-Pentanedione	85 ^c^	120 ^b^	122 ^b^	168 ^a^	1073	1073	
ket8	4-Methyl-3-penten-2-one	29	53	155	196	1133		1110–1159
ket9	2,4-Pentanedione	7	18	20	54	1162		1167–1230
ket10	2-Heptanone	45	43	46	32	1198	1190	
ket11	4-Methyl-2-heptanone	21	32	38	40	1219		1206–1224
ket12	6-Methyl-5-hepten-2-one	9	12	44	26	1350		1296–1368
ket13	2-Nonanone	45	34	29	8	1402	1382	
ket14	Acetophenone	142	138	109	133	1663		1600–1695
lac1	Butyrolactone	50	52	43	0	1641		1593–1673
lac2	delta-Hexalactone	14	11	9	4	1816		1751–1830
lac3	gamma-Heptalactone	118	83	80	5	1825		1755–1817
lac4	gamma-Nonalactone	140	102	71	2	2063		1981–2068
lac5	gamma-Decalactone	36	23	13	1	2176		2090–2185
aci1	Acetic acid	19	67	17	272	1459		1401–1485
aci2	3-Methylbutanoic acid	13 ^b^	23 ^b^	2 ^b^	197 ^a^	1679		1631–1707
aci3	Pentanoic acid	1	9	0	8	1747	1755	
aci4	Hexanoic acid	5	25	3	210	1859		1797–1889
aci5	Octanoic acid	1	9	1	20	2078		2011–2100
aci6	Benzoic acid	26 ^b^	46 ^ab^	49 ^ab^	85 ^a^	2415		2380–2457
alk1	Tetradecane	21 ^b^	28 ^b^	41 ^a^	41 ^a^	1405	1400	
alk2	Hexadecane	19	16	20	17	1602	1600	
alk3	Heptadecane	8	5	8	9	1700	1700	
alk4	Octadecane	16	14	24	20	1800	1800	
ben1	1,2,4-Trimethylbenzene	15	15	17	17	1285		1247–1333
phe1	Phenol	57	73	50	59	2024		1949–2037
oth1	3-Methylbutanenitrile	63^c^	74 ^b^	75 ^b^	90 ^a^	1121		1090–1144
oth2	Thiazole	20 ^c^	36 ^b^	40 ^b^	63 ^a^	1259		1210–1270

^1^ alc = alcohol, ald = aldehyde, est = ester, fur = furan, ket = ketone, lac = lactone, aci = organic acid, alk = alkane, ben = benzaldehyde, phe = phenol, oth = other; ^2^ Within each row, different letters (a–d) indicate a significant difference (*p* ≤ 0.05) in relative concentration between time points.
